# The Astrocyte Elevated Gene- 1 Expression and other Risk Factors for Different Varieties of Diabetic Optic Neuropathy in Egyptian Patients with Diabetic Retinopathy

**DOI:** 10.1007/s12031-025-02372-1

**Published:** 2025-06-19

**Authors:** Noha Rabie Bayomy, Sameh Mohamed Elgouhary, Eman Masoud Abd El Gayed, Karema Abdelhady Diab, Mai A. Kamel, Nashwa Mahmoud Mouhamed Muharram

**Affiliations:** 1 Department of Medical Biochemistry and Molecular Biology, Menoufia University, Shebin Elkom, Egypt; 2 Department of Medical Biochemistry and Molecular Biology, Benha National University, Obour, Egypt; 3 Department of Ophthalmology, Menoufia University, Shebin Elkom, Egypt; 4 Department of Clinical Pathology, National Liver Institute, Shebin Elkom, Egypt; 5 Department of Internal Medicine, Menoufia University, Shebin Elkom, Egypt

**Keywords:** *AEG-1*, MRNA expression, Diabetic optic neuropathy, PCR

## Abstract

To assess the role of the mRNA and protein expression of Astrocyte Elevated Gene- 1 (*AEG-1*) and other possible risk factors for diabetic optic neuropathy (DON) in Egyptian patients with diabetic retinopathy (DR). This case–control study constituted 450 diabetic patients divided into three groups: Group 1 included 150 DR patients with any sign of DON (diabetic papillopathy (DP), non-arteritic anterior ischemic optic neuropathy (AION), and optic atrophy (OA)). Group 2 included 150 DR patients without any sign of DON. Group 3 included 150 sex and age-matched diabetic patients without any sign of DR or DON. The expression level of *AEG-1* mRNA was assessed by real-time PCR. Serum AEG-1 was determined by ELISA. Multivariate logistic regression analysis was conducted to assess the DON risk factors. The mRNA expression of the *AEG-1* gene and AEG-1 protein levels were significantly increased (P < 0.001) in diabetic patients with (group 1) than without (group 2) DON. ROC curve analysis showed that the mRNA level of the AEG-1 gene exhibited a 70% specificity and a 79.33% diagnostic sensitivity. The diagnostic sensitivity of the serum concentration of AEG-1 was 84.67%, while the specificity was 70%. Multivariate regression analysis showed that the risk factors for DON were 2hpp blood sugar, HbA1c, serum cholesterol, *AEG-1* gene's mRNA expression, and AEG-1 protein. Serum cholesterol, the *AEG-1* gene's mRNA expression, and the AEG-1 protein are risk factors for all varieties of DON. While the blood glucose levels favour the development of DP, control of diabetes favours the occurrence of AION. Lastly, diabetes duration is more related to the presence of OA.

## Introduction

Diabetic retinopathy (DR), diabetic macular oedema, and diabetic optic neuropathy (DON) are the most prevalent vision-threatening complications in diabetics **(**Giuliari et al. 2011**)**.

The varieties of DON are diabetic papillopathy (DP), neovascularization at the optic disc (NVD), non-arteritic anterior ischemic optic neuropathy (AION), and optic atrophy (OA) (Ding et al. 2005).

According to Reddy et al. 2015**,** eyes with DR had an AION frequency of 30.1%. Diabetic papillopathy and AION are ischemic optic neuropathies. About 36% of diabetic patients with DP eventually progress to AION **(**Almog and Goldstein 2003**)**.

Diabetic papillopathy is self-limiting. It can be unilateral or bilateral and can occur in both DM types. The visual prognosis is usually good. However, some patients experience irreversible vision loss **(**Giuliari et al. 2011**)**. The common ocular manifestations of DP include mild blurring of vision, normal pupillary reaction, superficial dilated disc telangiectasia, an enlarged blind spot, unilateral or bilateral disc oedema, and disc hyper-fluorescence with late leakage in fundus fluorescein angiography (FFA) **(**Mallika et al. 2012**)**. Some patients with DP may present with no symptoms at all **(**Becker et al. 2024**)**. High haemoglobin A1c (HbA1c) levels and a reduced cup/disc ratio are risk factors for DP **(**Ostri et al. 2010**)**. Diabetic papillopathy may be considered a localized, DM-related optic disc vasculopathy. Due to temporary fluid leaking brought on by this vasculopathy, disc oedema develops. When there is persistent hyperglycemia and anoxia, the optic disc oedema can impair the axoplasmic flow and result in optic nerve toxicity **(**Hua et al. 2019**)**.

The second variant of optic neuropathy, nonarteritic anterior ischemic optic neuropathy, is a major contributor to diabetic adult vision loss **(**Hathaway et al. 2024). Its manifestations are acute loss of vision, optic disc swelling, relative afferent pupillary defect and visual field defects. It is not clear whether DP and AION are different aetiologic and diagnostic entities or whether DP is a mild form of AION **(**Huemer et al. 2022**).**

Prajapati and Dass, 2025 stated that DM can cause apoptosis of retinal ganglion cells leading to exacerbation of optic disc cupping and visual field changes. OCT parameters of ONH found that DM leads to reduced viability of RNFL,increased optic disc pallor as increase in CDR and parapapillary atrophy. Neuroretinal rim can be the important OCT parameter that can differentiate diabetes and other pathologies induced optic nerve damage.

The AEG-1 also referred to as metadherin (MTDH), lysine‐rich carcinoembryonic antigen-related cell adhesion molecule 1 (CEACAM1) co-isolated (LYRIC), and 3D3. AEG-1 is the primary activator of inflammation through the transcription factor NF-κB, which is the master regulator of inflammation, mediating the production of pro-inflammatory cytokines. Astrocyte elevated gene-1 is a lipopolysaccharide (LPS) responsive gene that, via NF-κB activation, is crucial for the synthesis of prostaglandin E2 (PGE2) when induced by LPS **(**Khuda et al. 2009**)**. AEG-1 plays a role in facilitating reactive astrogliosis **(**Vartak-Sharma and Ghorpade 2012**)**.

The association between *AEG-1* mRNA expression and its serum protein levels with DON susceptibility and other systemic and ocular risk factors of DON in Egyptian diabetic patients was assessed. This study is, to our knowledge, the inaugural investigation of this susceptibility.

## Subjects and Methods

### Study Participants and Setting

**Fig. 1:**
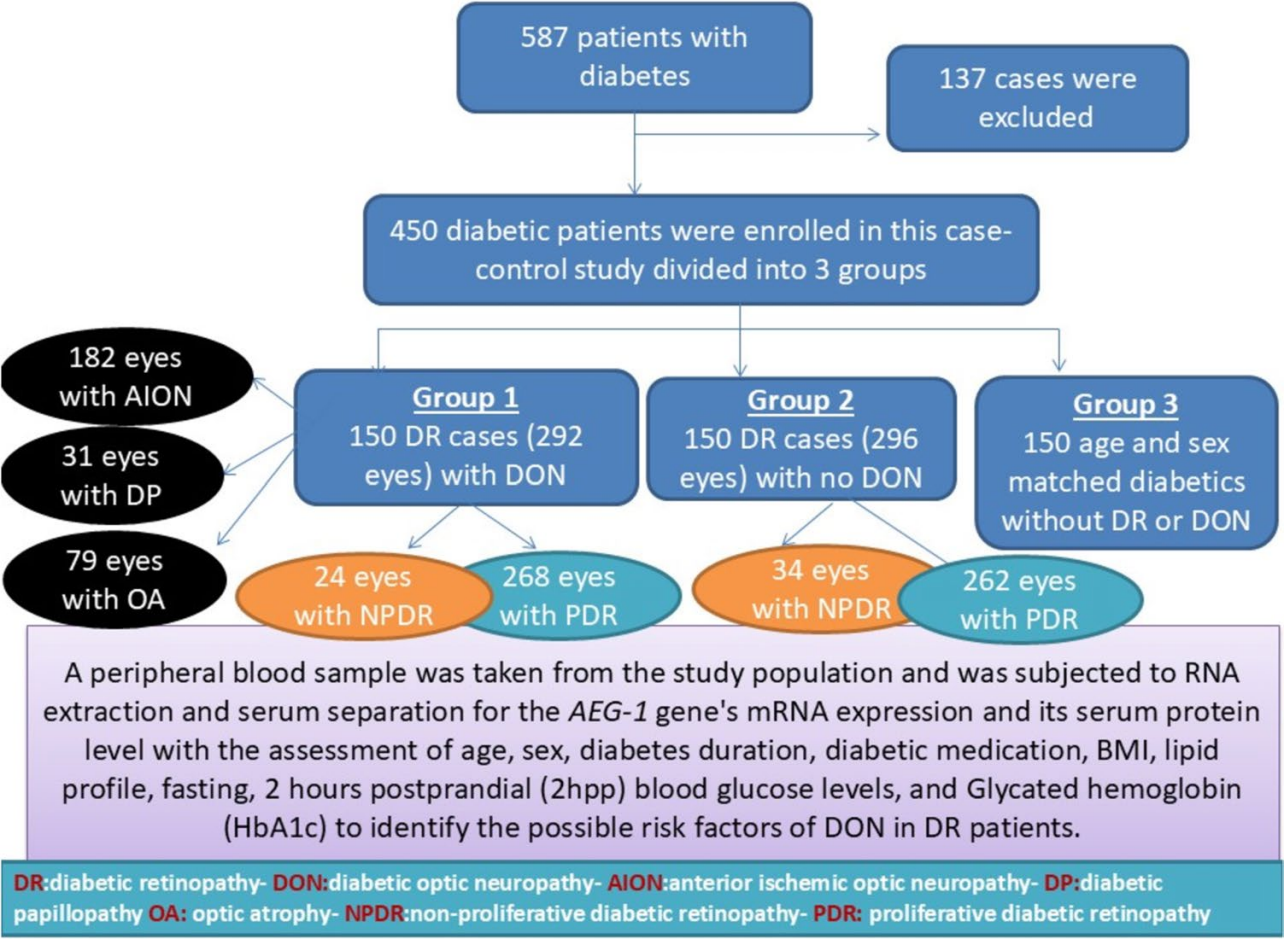
The study design

The study design was summarized in Fig. 1. The study was given the 72020BIO approval number by the Menoufia University Faculty of Medicine's ethics committee and adhered to the principles of the Declaration of Helsinki. After discussing the study's objectives and methods, the participants'oral and written informed consents were gained. This study was conducted at the Medical Biochemistry and Molecular Biology department in association with the Internal Medicine and Ophthalmology Departments and the Central Laboratory, Menoufia University, and Hospitals between November 2020 and November 2022.

This case–control study constituted 450 diabetic patients categorized into three groups: Group 1 comprised 150 DR patients with any sign of DON (DP, AION, or OA). Group 2 included 150 DR patients without any sign of DON. Group 3 included 150 sex and age-matched diabetic patients without any sign of DR or DON.

Every enrolled patient underwent: 1) Thorough history-taking including the duration and the medication of diabetes, 2) Physical examination, including anthropometric measurements. By dividing body weight (kilograms) by the square of height (meters), the body mass index (BMI) was calculated manually, 3) Measurement of serum lipid profile [total cholesterol (TC), triglycerides (TG), high-density lipoprotein cholesterol (HDLc)], and low-density lipoprotein cholesterol (LDLc) 4) Measurement of Fasting blood sugar (FBG) and two hours postprandial (2hpp) blood glucose and Glycated haemoglobin (HbA1c) 5) Measurement of the *AEG1* gene's mRNA expression and its serum protein level.

We used the International Clinical Diabetic Retinopathy Disease Severity Scales for grading DR **(**Wilkinson et al. 2003**)**. Proliferative diabetic retinopathy (PDR) is different from non-proliferative diabetic retinopathy (NPDR) by the existence of neovascularization (NVE) at the optic disc (NVD) or elsewhere, the presence of vitreous haemorrhage or tractional retinal detachment. Diabetic optic neuropathy was categorized according to the study conducted by the Zhongshan Ophthalmic Center **(**Ding et al. 2005**)** and the study of Wang et al. 2017**.** In DP, the optic disc exhibits non-specific hyperemic swelling or prominent dilated vessels that resemble neovascularization on the disc surface. In AION, the optic nerve head shows hyperaemic diffuse or segmental oedema with splinter peripapillary haemorrhages. In OA, the optic nerve head is pale with the loss of its normal pink colour.

We excluded patients with (1) background of eye procedures (such as anti-vascular endothelial growth factor injection, laser photocoagulation, or vitrectomy); (2) previous ocular surgeries; and (3) patients with primary optic atrophy (OA) or OA secondary to retinal diseases, e.g., retinitis pigmentosa or glaucomatous OA.

### Biochemical Analyses

By sterile vein puncture, about 10 millilitres of venous blood was taken from every subject. The first 2 ml of blood was transferred into an EDTA tube: which was used for quantitative colorimetric determination of HA1c as a percentage of the total hemoglobin using kits supplied by Teco Diagnostics, USA. Following 8 h of fasting, one millilitre of blood was drawn into a tube with sodium fluoride to measure FBG. After two hours, a further 1 ml blood sample was collected for 2hpp blood glucose. Using the Spinreact kit from SPAIN, blood glucose was determined by the enzymatic colorimetric method **(**Attia et al. 2014**)**. Then, after 12 h of overnight fasting, four millilitres of blood was placed into a clear tube, permitted to coagulate at 37 °C for 10 min and then centrifuged. The transparent serum supernatant was gathered and stored at −80 °C until the determination of serum AEG-1 protein, serum HDL, TC, TG, and LDL (El-Hefnawy et al. 2018), (Rifai and Warnick 2006). Using kits from Spinreact (SPAIN), the enzymatic colorimetric approach was used to determine the serum TC and TG levels with cataloge numbers TK41021 and TK41030 respectively. While HDL-c was assessed using a colorimetric kit from"Human Diagnostics mbH, GERMANY"with cataloge No:10084. The Friedewald equation computed LDL-c using TG, HDL-c, and TC (Dorak 2000). Serum AEG-1 was determined by ELISA using the MyBiosource® human Metadherin (AEG-1) ELISA kit, USA, with catalog No: MBS4501182.

### Estimation of AEG-1 Gene's mRNA Expression by Real-Time qPCR

#### Total RNA Extraction

In an EDTA-containing tube, the last 2 ml of blood was added to estimate the *AEG1* gene's mRNA expression by RT-qPCR. According to the manual guide, the Whole Blood RNA Purification Mini Kit from Thermo Scientific Gene JET was used to isolate total RNAs from peripheral blood WBCs.

The RNA concentration was calculated using Beer-Lambert's formula with the Nanophotometer N60, Implen-Germany to evaluate its 260 nm and 280 nm absorbances (A260 and A280, respectively). A260/A280 is the ratio that estimates RNA purity. The 260/280 ratio of the RNA extract was between 1.8 and 2, which is accepted. Until the following step, the RNA extract was kept at −80 C.

The first-step PCR of complementary DNA (cDNA) synthesis was performed using cDNA Reverse Transcription Kits with High Capacity from Applied Biosystems, USA.

The second step in real-time PCR was carried out utilizing Applied Biosystems QuantiTect SYBR Green PCR Kit with QuantiTect primers. For the assessment of human *AEG-1* mRNA levels, the subsequent forward and reverse primers were utilized.: 5′-ACGACCTGGCCTTGCTGAAGAATCT-3′ and 5′-CGGTTGTAAGTTGCTCGGTGGTAA-3′, respectively. Forward and reverse primers for *glyceraldehyde-3-phosphate dehydrogenase* (*GAPDH*) were: 5′-ATGGGGAAGGTGAAGGTCGGAGTC-3′ and 5′-GCTGATGATCTTGAGGCTGTTGTC-3′, respectively.

**Fig. 2:**
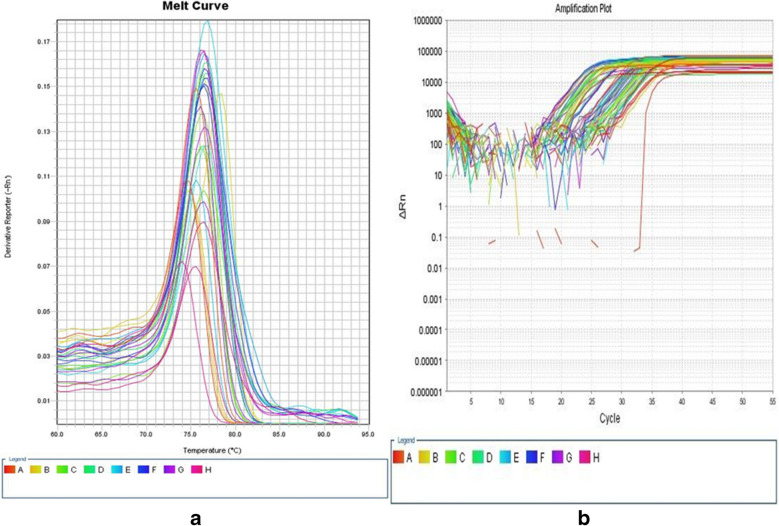
**a** and **b**: Amplification plot (**a**) and melting curve (**b**) of *AEG-1* mRNA gene expression

Each reaction mixture for each gene was conducted at a total volume of 20 µL., ten μL SYBR Green 2 × QuantiTect PCR Master Mix, one μL forward primer, one μL reverse primer, and variable amounts of cDNA and RNase-free H2O according to the initial RNA concentration to ensure a 100 ng of cDNA per reaction. PCR was conducted under the following conditions: Three minutes of incubation at 94 °C, followed by 60 cycles (at 94 °C for 30 s of denaturation, at 55 °C for 40 s of annealing, and at 72 °C for 31 s of extension) (Fig. 2a). Gene expression's relative quantitation (RQ) was performed using the Comparative ΔΔCt method **(**Wang et al. 2017**)**. The mRNA level of the housekeeping gene *GAPDH* was used to normalize the amount of *AEG-1* mRNA. A melting curve was carried out to verify the specificity of the amplification and the absence of primer dimers **(**Fig. 2b**)**.

### Statistical Analysis

The data were analyzed by the IBM SPSS program version 20.0 (Armonk, NY: IBM Corp). The Kolmogorov–Smirnov test was utilized to confirm the normality of the variables'distribution. Using the Chi-square test, comparisons between groups for categorical variables were evaluated. ANOVA was used to compare the three groups under study, and a Post Hoc test (Tukey) was then utilized to compare two groups at once. For quantitative variables with abnormal distribution, the Kruskal–Wallis test was utilized. The Post Hoc test (Dunn's for multiple comparisons test) for pairwise comparison came next. Mann–Whitney test compared two groups with not normally distributed quantitative variables. The Spearman coefficient correlates the quantitative variables together. Binary Logistic Regression detects the most independent factor affecting DR with DON. The diagnostic efficacy of the markers was evaluated using the receiver operating characteristic curve (ROC), a performance of more than 50% is considered acceptable, and the best result for the test is an area of about 100%. The 5% level was used to determine the data's significance.

## Results

### Clinical Data and Demographics of the Study Participants (Table 1)

**Table 1:** Clinical data and demographics of the study participants

Demographic and clinical data	Group 1 (*n* = 150)	Group 2 (*n* = 150)	Group 3 (*n* = 150)	Test of Sig	*P*
Age (years)					
Mean ± SD	53.81 ± 7.58	53.05 ± 7.26	54.05 ± 8.05	F = 0.701	0.497
Median (Min. – Max.)	52 (41–68)	53 (41–69)	52 (41–68)		
Sex					
Male	78 (52.0%)	88 (58.7%)	80 (53.3%)	χ^2^ = 1.506	0.471
Female	72 (48.0%)	62 (41.3%)	70 (46.7%)		
BMI (kg/m^2^)					
Mean ± SD	28.86 ± 3.23	29.22 ± 3.35	20.91 ± 1.73	F = 403.42^*^	< 0.001^*^
Median (Min. – Max.)	29.0(22.0–34.0)	29.1(22.5–36.7)	21.0(18.0–26.0)		
Significance between groups	p_1_ = 0.522,p_2_ < 0.001^*^,p_3_ < 0.001^*^				
Systolic BP					
Mean ± SD	141.7 ± 11.0	139.9 ± 9.85	112.3 ± 9.35	F = 400.92^*^	< 0.001^*^
Median (Min. – Max.)	140 (110–170)	140 (110–170)	120 (100–120)		
Significance between groups	p_1_ = 0.298,p_2_ < 0.001^*^,p_3_ < 0.001^*^				
Diastolic BP					
Mean ± SD	92.67 ± 6.87	92.87 ± 7.60	76.73 ± 7.49	F = 239.42^*^	< 0.001^*^
Median (Min. – Max.)	90 (70–110)	90 (70–110)	70 (70–90)		
Significance between groups	p_1_ = 0.970,p_2_ < 0.001^*^,p_3_ < 0.001^*^				
Diabetic medication of the enrolled patients					
Oral antidiabetics	133 (88.7%)	141 (94%)	150 (100.0%)	χ^2^ = 17.716 303.71^*^	< 0.001^*^
Insulin	17 (11.3%)	9 (6%)	0 (0.0%)		
Duration of diabetes (in years)					
Mean ± SD	14.647 ± 3.589	14.307 ± 2.859	10.447 ± 1.065	F = 100.407^*^	< 0.001^*^
Median (Min. – Max.)	14.50 (13–21)	14.00 (10–18)	10.75 (9–12)		
Significance between groups	p_1_ = 0.970,p_2_ < 0.001^*^, p_3_	< 0.001^*^			
Grading of DR					
NPDR PDR	24 (8.2%) 268 (91.8%)	34 (11.5%) 262 (88.5%)	-	χ^2^ = 2.453	0.117
			-		

BMI: body mass index BP: blood pressure F: F for ANOVA test, SD: Standard deviation χ^2^: Chi-square test. Pairwise comparison bet. Each pair of groups was analyzed using the Post Hoc Test (Tukey). p: p-value for the comparison of the three examined groups; p1: p-value for the comparison between Group 1 and Group 2; p2: p-value for the comparison between Group 1 and Group 3; p3: p-value for the comparison between Group 2 and Group 3. *: Statistically significant at p ≤ 0.05.

The age and sex did not exhibit significant differences across the three groups, (*p* = 0.497 and *p* = 0.471, respectively). In group 1, there were 182 eyes (62.4%) with AION, 31 eyes with DP (10.6%), and 79 eyes (27%) with OA. There was a significant difference regarding insulin treatment for DM between the diabetic patients with (11.3%) and without (6%) DON. There were 292 eyes in group 1 and 296 eyes in group 2. In group 1, there were 24 eyes (8.2%) with NPDR and 268 eyes (91.8%) with PDR. In group 2, there were 34 eyes (11.5%) with NPDR and 262 eyes (88.5%) with PDR.

### The Laboratory Data of the Enrolled Participants (Table 2)

**Table 2:** The laboratory data of the enrolled participants

Laboratory data	Group 1 (*n* = 150)	Group 2 (*n* = 150)	Group 3 (*n* = 150)	F	*P*
FBG (mg/dl)					
Mean ± SD	242.71 ± 33.32	224.16 ± 37.71	105.13 ± 8.69	498.992^*^	< 0.001^*^
Median (Min. – Max.)	242 (160–380)	223 (148–347)	102 (90–113)		
Significance between groups	p_1_ < 0.001^*^,p_2_ < 0.001^*^,p_3_ < 0.001^*^				
2hpp blood glucose (mg/dl)					
Mean ± SD	281.11 ± 24.61	244.31 ± 46.23	124.56 ± 9.16	595.172^*^	< 0.001^*^
Median (Min. – Max.)	278 (240 - 351)	238 (174 - 452)	122.0 (108–140)		
Significance between groups	p_1_ < 0.001^*^,p_2_ < 0.001^*^,p_3_ < 0.001^*^				
HbA1c					
Mean ± SD	8.39 ± 0.77	8.23 ± 0.63	5.23 ± 0.77	895.698^*^	< 0.001^*^
Median (Min. – Max.)	8.50 (6.10 - 9.80)	8.20 (6.30 - 9.80)	5.10 (3.70–6.80)		
Significance between groups	p_1_ = 0.114,p_2_ < 0.001^*^,p_3_ < 0.001^*^				
HDL-c (mg/dl)					
Mean ± SD	33.95 ± 5.37	38.85 ± 5.16	46.51 ± 5.59	208.13^*^	< 0.001^*^
Median (Min. – Max.)	33 (28–50)	38 (33–56)	48 (28–50)		
Significance between groups	p_1_ < 0.001^*^,p_2_ < 0.001^*^,p_3_ < 0.001^*^				
TGs (mg/dl)					
Mean ± SD	177.6 ± 18.99	179.1 ± 19.13	93.33 ± 4.09	1458.0^*^	< 0.001^*^
Median (Min. – Max.)	172 (150–230)	173 (151–231)	95 (83–98)		
Significance between groups	p_1_ = 0.690,p_2_ < 0.001^*^,p_3_ < 0.001^*^				
TC (mg/dl)					
Mean ± SD	221.7 ± 26.9	213.3 ± 26.91	170.3 ± 9.72	222.01^*^	< 0.001^*^
Median (Min. – Max.)	222 (182–287)	214 (174–278)	169 (155–186)		
Significance between groups	p_1_ = 0.004^*^,p_2_ < 0.001^*^,p_3_ < 0.001^*^				
LDL-c (mg/dl)					
Mean ± SD	151.64 ± 23.26	149.65 ± 23.25	103.1 ± 9.13	291.225^*^	< 0.001^*^
Median (Min. – Max.)	147.1 (124.3 - 289.3)	145.5 (122.8 - 286.1)	104.0 (88.0–118.0)		
Significance between groups	p_1_ = 0.655,p_2_ < 0.001^*^,p_3_ < 0.001^*^				

FBG: fasting blood glucose 2hpp: two hours postprandial HbA1c: glycated haemoglobin TC: total cholesterol TGs: triglycerides LDL-c: low-density lipoprotein-cholesterol HDL-c: high-density lipoprotein-cholesterol. F: F for ANOVA test, SD: Standard deviation, Every two groups were compared pairwise using the Post Hoc Test (Tukey)

The p-values for the three groups under study are as follows: p1 corresponds to the comparison between Group 1 and Group 2, p2 to the comparison between Group 1 and Group 3, and p3 to the comparison between Group 2 and Group 3. *: At p < 0.05, it was statistically significant

Compared to the other two groups, group 1 showed a significant decrease in HDL-c levels and a significant increase in FBG, 2 h pp blood sugar, HbA1C, LDL-c and TC levels.

### The AEG-1 mRNA Expression and its Protein Levels in the Studied Groups (Table 3)

**Table 3:** The AEG-1 mRNA expression and its protein levels in the studied groups

	Group 1 (*n* = 150)	Group 2 (*n* = 150)	Group 3 (*n* = 150)	*H*	*p*
AEG-1 mRNA					
Mean ± SD	53.10 ± 25.75	24.64 ± 16.08	7.67 ± 14.04	228.640^*^	< 0.001^*^
Median (Min. – Max.)	53.17(0.75 - 109.01)	22.50(0.75 - 74.60)	2.45(0.05–73.35)		
Significance between groups	p_1_ < 0.001^*^,p_2_ < 0.001^*^,p_3_ < 0.001^*^				
AEG-1 Protein (ng/ml)					
Mean ± SD	6.76 ± 1.96	4.21 ± 1.40	2.37 ± 1.81	233.604^*^	< 0.001^*^
Median (Min. – Max.)	6.90 (2.01 - 10.0)	4.01 (1.71 - 8.12)	1.92 (0.06–8.14)		
Significance between groups	p_1_ < 0.001^*^,p_2_ < 0.001^*^,p_3_ < 0.001^*^				

AEG-1: Astrocyte Elevated Gene-1 SD: Standard deviation H: H for Kruskal Wallis test, The Post Hoc Test (Dunn's for multiple comparisons test) was used to compare each two groups pairwise: p: p-value for comparing the three groups under study, p1: p-value for comparing Group 1 with Group 2, p2: p-value for comparing Group 1 with Group 3, and p3: p-value for comparing Group 2 with Group 3.*: Statistical significance was determined at p ≤ 0.05

The mRNA expression of the *AEG-1* gene and its protein levels were significantly increased (P < 0.001) in groups 1 and 2 compared to group 3. Also, The mRNA expression of the *AEG-1* gene and its protein levels were significantly increased (P < 0.001) in diabetic patients with DON (group 1) than without DON (group 2).

### Correlating the AEG-1 Gene's Expression Levels to Demographics, Clinical Data, and Laboratory Data in Groups 1 and 2 (Table 4)

**Table 4:** Correlating the *AEG-1* gene's expression levels to demographics, clinical data and laboratory data in groups 1 and 2

	Group 1 (n = 150)				Group 2 (n = 150)			
	AEG-1 mRNA		AEG-1 Protein (ng/ml)		AEG-1 mRNA		AEG-1 Protein (ng/ml)	
	r_s_	P	r_s_	P	r_s_	p	r_s_	p
Age (years)	−0.123	0.135	−0.060	0.463	−0.179	0.028^*^	−0.218	0.007^*^
BMI (kg/m^2^)	0.651	< 0.001^*^	0.655	< 0.001^*^	0.280	0.001^*^	0.390	< 0.001^*^
Systolic BP	0.077	0.348	0.030	0.717	−0.132	0.107	−0.185	0.024^*^
Diastolic BP	0.128	0.119	0.018	0.823	−0.158	0.053	−0.159	0.052
FBG (mg/dl)	0.959	< 0.001^*^	0.802	< 0.001^*^	0.153	0.062	−0.037	0.656
2hpp blood glucose (mg/dl)	0.952	< 0.001^*^	0.804	< 0.001^*^	0.221	0.007^*^	0.162	0.047^*^
HbA1c	0.821	< 0.001^*^	0.703	< 0.001^*^	0.317	< 0.001^*^	0.234	0.004^*^
HDL-c (mg/dl)	−0.431	< 0.001^*^	−0.330	< 0.001^*^	−0.263	0.001^*^	−0.150	0.067
TGs (mg/dl)	0.726	< 0.001^*^	0.626	< 0.001^*^	0.351	< 0.001^*^	0.382	< 0.001^*^
T. Cholesterol (mg/dl)	0.433	< 0.001^*^	0.379	< 0.001^*^	0.098	0.233	0.184	0.024^*^
LDL-c (mg/dl)	0.907	< 0.001^*^	0.831	< 0.001^*^	0.538	< 0.001^*^	0.621	< 0.001^*^

In group 1, there was a significant positive correlation between the *AEG-1* gene's mRNA expression level and AEG-1 protein with body mass index (BMI), FBG, 2 h postprandial (2 h.p.p) blood glucose, glycated haemoglobin (HbA1c), lipid profile [serum triglycerides (TG) total cholesterol (TC) and low-density lipoprotein (LDLc)], in contrast to a negative correlation with high-density lipoproteins, HDLc (P < 0.001). In group 2, the *AEG-1* gene's mRNA expression level and AEG-1 protein showed a significant positive correlation with BMI, 2 h.p.p. blood glucose, HbA1c, serum TG and LDL-c) while the *AEG-1* gene's mRNA expression showed a negative correlation with HDL-c (P < 0.001).

### The Diagnostic Value of the AEG-1 mRNA and its Serum Protein in Distinguishing the DR Patients with DON from those Without DON (Fig. 3 and Table 5)

**Fig. 3:**
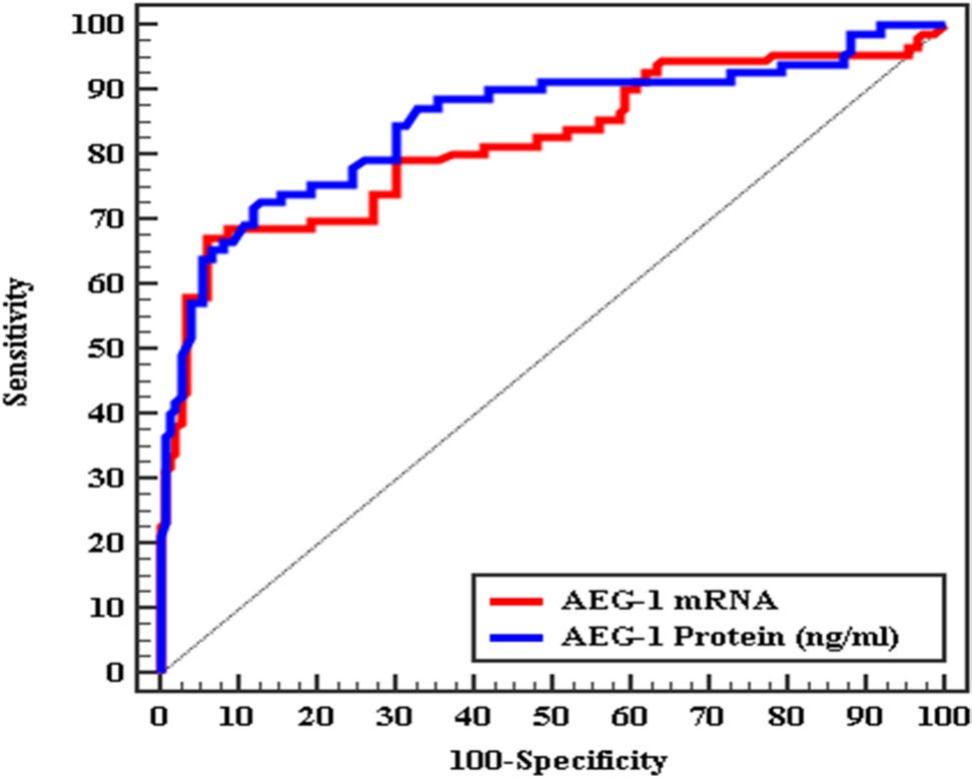
ROC curve for AEG-1 mRNA and AEG-1 Protein to discriminate diabetic retinopathy patients with DON (group 1) from those without DON (group 2)

**Table 5:** The diagnostic value of the AEG-1 mRNA and its serum protein in distinguishing the DR patients with DON from those without DON

	AUC	P	95% CI	Cut off^#^	Sensitivity	Specificity	PPV	NPV
AEG-1 mRNA	0.820	< 0.001^*^	0.771 - 0.869	> 33.84	79.33	70.0	72.6	77.2
AEG-1 Protein	0.851	< 0.001^*^	0.806 - 0.896	> 4.93	84.67	70.0	73.8	82.0

AUC: Area Under a CurveCI: Confidence Intervals NPV: Negative predictive value PPV: Positive predictive value *: Statistically significant at p ≤ 0.05

The ideal cut-off point for the *AEG-1* gene's mRNA expression is > 33.84 ng/ml; at this level, the mRNA expression of the *AEG-1* gene had a diagnostic sensitivity of 79.33%, a specificity of 70%, a PPV of 72.6%, and NPV of 77.2%. The ideal cut-off point for the AEG-1 serum protein level is > 4.93 ng/ml; at which, the AEG-1 serum concentration has a diagnostic sensitivity of 84.67%, a specificity of 70%, a PPV of 73.8%, NPV of 82%.

### Multivariate Logistic Regression for Risk Factors for the Different Varieties of DON (Table 6)

**Table 6:** Multivariate Logistic regression for risk factors for the different varieties of DON

DON type	OR (95%C.I)	P
DP		
BMI (kg/m^2^)	0.803 (0.613–1.050)	0.109
Diabetes duration	0.850 (0.716–1.009)	0.063
FBG (mg/dl)	1.053 (1.016–1.092)	0.005^*^
2hpp (mg/dl)	1.042 (1.005 - 1.080)	0.025^*^
HbA1c	1.678 (1.216 - 1.982)	0.034^*^
HDL-c (mg/dl)	0.506 (0.325 - 0.788)	0.003^*^
T. Cholesterol (mg/dl)	1.040 (1.018 - 1.063)	< 0.001*
TGs (mg/dl)	0.986 (0.963 - 1.010)	0.241
AEG-1 mRNA	1.165 (1.077 - 1.260)	< 0.001*
AEG-1 Protein (ng/ml)	2.391 (1.372 - 4.169)	0.002*
AION		
BMI (kg/m^2^)	1.155 (1.003–1.330)	0.045*
Diabetes duration	1.054 (0.994–1.117)	0.077
FBG (mg/dl)	1.002 (0.972–1.033)	0.914
2hpp (mg/dl)	1.057 (0.998 - 1.119)	0.060
HbA1c	1.351 (1.072 - 1.704)	0.011^*^
HDL-c (mg/dl)	0.341 (0.141 - 0.823)	0.017^*^
T. Cholesterol (mg/dl)	1.209 (1.102 - 1.327)	< 0.001*
TGs (mg/dl)	1.119 (1.017 −1.230)	0.020*
AEG-1 mRNA	1.239 (1.107 - 1.386)	< 0.001*
AEG-1 Protein (ng/ml)	2.559 (1.442 - 4.541)	0.001*
Optic Atrophy		
BMI (kg/m^2^)	0.894 (0.781–1.022)	0.102
Diabetes duration	2.001 (1.265–3.164)	0.003*
FBG (mg/dl)	0.973 (0.946–1.000)	0.053
2hpp (mg/dl)	1.033 (0.977 - 1.093)	0.248
HbA1c	2.034 (0.877 - 4.722)	0.098
HDL-c (mg/dl)	0.833 (0.712 - 0.974)	0.022^*^
T. Cholesterol (mg/dl)	1.176 (1.042 - 1.328)	0.003*
TGs (mg/dl)	1.103 (0.966 - 1.259)	0.147
AEG-1 mRNA	1.166 (1.107 - 1.229)	< 0.001*
AEG-1 Protein (ng/ml)	2.239 (1.395 - 3.591)	0.001*

FBG: fasting blood glucose 2hpp: two hours postprandial HbA1c: Glycated hemoglobin HDL-c: high-density lipoprotein-cholesterol TGs: triglycerides T. Cholesterol: total cholesterol AEG-1: Astrocyte Elevated Gene- 1 OR: Odd's ratioCI: Confidence interval LL: Lower limit UL: Upper Limit

#: All variables with p < 0.05 were included in the multivariate analysis *: Statistically significant at p ≤ 0.05

Regarding DP, multivariate regression analysis showed that the risk factors for DP were FBG (OR (95%C.I); 1.053 (1.016 - 1.092)), 2hpp blood sugar (OR (95%C.I); 1.042 (1.005 - 1.080)), HbA1c (OR (95%C.I); 1.678 (1.216 - 1.982)), serum cholesterol (OR (95%C.I); 1.040 (1.018 - 1.063)), AEG-1 gene's mRNA expression (OR (95%C.I); 1.165 (1.077 - 1.260)) and AEG-1 protein (OR (95%C.I); 2.391 (1.372 - 4.169)), while HDL-c was a protective factor (OR (95%C.I); 0.506(0.325 - 0.788)). Regarding AION, multivariate regression analysis showed that the risk factors for AION were HbA1c (OR (95%C.I); 1.351 (1.072 - 1.704)), serum cholesterol (OR (95%C.I); 1.209 (1.102 - 1.327)), serum triglycerides (OR (95%C.I); 1.119 (1.017 −1.230)), AEG-1 gene's mRNA expression (OR (95%C.I); 1.239 (1.107 - 1.386)) and AEG-1 protein (OR (95%C.I); 2.559 (1.442 - 4.541), while HDL-c was a protective factor (OR (95%C.I); 0.341 (0.141 - 0.823)). Regarding optic atrophy, multivariate regression analysis showed that the risk factors for optic atrophy were diabetes duration (OR (95%C.I); 2.001 (1.265–3.164)), serum cholesterol (OR (95%C.I); 1.176 (1.042 - 1.328)), AEG-1 gene's mRNA expression (OR (95%C.I); 1.166 (1.107 - 1.229)) and AEG-1 protein (OR (95%C.I); 2.239 (1.395 - 3.591)), while HDL-c was a protective factor (OR (95%C.I); 0.833 (0.712 - 0.974)).

### Multivariate Logistic Regression for Risk Factors Affecting the Severity of DR (Table 7)

**Table 7:** Multivariate Logistic regression for risk factors affecting the severity of DR

Severity of DR (NPDR)	**OR (95%C.I)**	**P**
BMI (kg/m^2^)	2.006 (1.269–3.170)	0.003*
Diabetes duration	0.974 (0.878–1.081)	0.619
FBG (mg/dl)	1.231 (1.096–1.382)	< 0.001*
2hpp (mg/dl)	1.356 (1.118 - 1.644)	0.002*
HbA1c	1.139(1.007 - 1.288)	0.038^*^
HDL-c (mg/dl)	0.689 (0.551 - 0.861)	0.001*
T. Cholesterol (mg/dl)	0.986 (0.951 - 1.023)	0.451
TGs (mg/dl)	0.972 (0.906 - 1.043)	0.437
AEG-1 mRNA	0.973 (0.878 - 1.080)	0.609
AEG-1 Protein (ng/ml)	1.789 (0.837 - 3.824)	0.134

FBG: fasting blood glucose 2hpp: two hours postprandial HbA1c: Glycated haemoglobin HDL-c: high-density lipoprotein-cholesterol T. Cholesterol: total cholesterol AEG-1: Astrocyte Elevated Gene- 1 OR: Odd's ratioCI: Confidence interval LL: Lower limit UL: Upper Limit

#: All variables with p < 0.05 were included in the multivariate analysis *: Statistically significant at p ≤ 0.05

Multivariate regression analysis showed that the risk factors affecting the severity of DR were FBG (OR (95%C.I); 1.231 (1.096–1.382)), 2hpp blood sugar (OR (95%C.I); 1.356 (1.118 - 1.644)) and HbA1c (OR (95%C.I); 1.139(1.007 - 1.288)), while HDL-c was a protective factor (OR (95%C.I); 0.689 (0.551 - 0.861)).

## Discussion

The microangiopathy associated with chronic hyperglycemia is linked with the failure of many organs, especially the nerves, eyes, and kidneys **(**VinodMahato et al. 2011**)**. AEG-1 leads to the secretion of many inflammatory mediators like tumour necrosis factor-α, necrosis factor-κB complex and increasing levels of Toll-like receptor 4 **(**Wang and Seki 2018**).** The progression of diabetes involves an interplay between metabolism and immunity causing the release of various types of immune cells (Bhargava and Lee 2012**).**

There have been numerous investigations of the glial and neuronal cell types that are changed in the retina during diabetes using animal models of hyperglycemia. Impairments in cone and rod photoreceptor function have been identified in retinal neurons **(**Phipps et al. 2004, 2006; Li et al. 2002**).** Also, alterations in downstream neurons, including ganglion, bipolar, and amacrine cells, had been detected. Significant metabolic pathway abnormalities have been linked to these functional neuronal impairments, which could ultimately increase diabetic-induced neuronal death **(**Phipps et al. 2004; Li et al. 2002; Hancock and Kraft 2004**).**

In the current study, The mRNA expression of the *AEG-1* gene and AEG-1 protein levels were significantly increased in diabetic patients with (groups 1) and without (group 2) DON compared to diabetics without DR or DON (group 3). Also, The mRNA expression of the *AEG-1* gene and AEG-1 protein levels were significantly increased in diabetic patients with than without DON. According to Selim et al. 2019, newly diagnosed type II diabetic patients have considerably higher peripheral blood monocyte AEG-1 gene expression than control participants.

This series revealed that the *AEG-1* gene's mRNA level had a diagnostic sensitivity of 79.33% and a specificity of 70%. Also, the diagnostic sensitivity of the AEG-1 protein serum concentration was 84.67%, and the specificity was 70%.

This study showed that serum cholesterol, the *AEG-1* gene's mRNA expression and AEG-1 protein are common risk factors for all varieties of DON while the blood glucose levels (FBG and 2hpp) favour the development of DP, control of diabetes (HbA1c) favours the occurrence of AION, lastly diabetes duration is more related to the presence of optic atrophy. We explain that DP is acute and may be a transient condition, so it is related more to the rapid change of blood glucose levels than the diabetic control or duration. If the blood glucose is then controlled, the DP will be a transient self-limiting condition, and if it is poorly controlled, the DP will progress to AION. This is supported by our finding that AION is related more to the control of diabetes (HbA1c) and also by the findings of Almog and Goldstein 2003 that 36% of diabetic patients with DP eventually progress to AION. Optic atrophy is a chronic condition, so it is more related to diabetes duration than other risk factors of DON.

Optic neuropathy may be caused by poor metabolic control and a sudden tightening of blood sugar (for example, during pregnancy, and after insulin therapy) (Bayraktar et al. 2002). Plasma HbA1c and HDL concentrations could affect how DON develops. Hua et al. 2019 findings showed that an increase in age, diabetes duration, elevated systolic blood pressure and DR severity were associated with increased risk of developing DP, NVD, and OA. Also, they stated that HbA1c was an important factor for developing NVD, AION, and OA. In a nationwide cohort study by Lee et al. 2023, they stated that the risk of optic neuritis in diabetic patients was increased with the duration of DM and high glucose levels. Accordingly, any efforts to regulate blood glucose levels and prevent DM progression could reduce the risk of optic neuritis. Agreeing with our findings, Hu et al., 2019 stated that patients with DON had considerably lower HDL levels than diabetics without DON. According to Sharma et al. 2017, hyperlipidemia poses a risk for AION in diabetic patients.

This study had some limitations. First, this study's sample size was quite small; however, this pilot study can serve as a useful starting point for future research with a larger patient population. Second, because serum protein and mRNA expression assessments were carried out on peripheral blood, caution should be exercised when extrapolating these findings to ocular tissues. It has been suggested that peripheral markers mimic the same disease processes in neural tissues. As a result, blood anomalies may be disease pathology markers. Third, for the diagnostic value of AEG-1 mRNA and its serum protein; the sensitivity and specificity are below 80%. This level of sensitivity and specificity may be quite acceptable for a pilot study.

## Data Availability

No datasets were generated or analysed during the current study.
